# Active surveillance vs. surgery in low‐risk papillary thyroid microcarcinoma patients and the risk of loss to follow‐up

**DOI:** 10.1002/cam4.70123

**Published:** 2024-08-28

**Authors:** Yoshiyuki Saito, Kenichi Matsuzu, Hiroshi Takami, Ai Matsui, Yoko Kuga, Ryoji Ohara, Kana Yoshioka, Chie Masaki, Junko Akaishi, Kiyomi Y. Hames, Ritsuko Okamura, Chisato Tomoda, Akifumi Suzuki, Wataru Kitagawa, Mitsuji Nagahama, Kiminori Sugino, Koichi Ito

**Affiliations:** ^1^ Department of Surgery Ito Hospital Tokyo Japan

**Keywords:** active surveillance, adherence, loss to follow up, papillary thyroid microcarcinoma, thyroidectomy

## Abstract

**Background:**

Papillary thyroid microcarcinoma (PTMC) management has evolved, with active surveillance (AS) gaining prominence as a management option. However, a key concern for both clinicians and patients is the potential for patient loss to follow‐up during AS.

**Aims:**

This study aimed to determine adherence and loss‐to‐follow‐up rates in low‐risk PTMC patients undergoing AS versus surgical intervention, in order to gain insights into clinical pathways and safety profiles.

**Materials and Methods:**

This cohort study analyzed the 2016 data from a single registered institution of Japan's public National Cancer Registry.

**Results:**

We identified and retrospectively analyzed the cases of 327 patients diagnosed with low‐risk PTMC; 227 patients chose to undergo AS while the other 100 underwent PTMC surgery. Main outcomes were the adherence rate and loss‐to‐follow‐up rate of each group, factors influencing discontinuation, and safety considerations. The rate of AS adoption was substantial in the complete series of 327 low‐risk PTMC patients (69.4%). There was a significantly higher loss‐to‐follow‐up rate at 5 years in the AS group (28.6%) compared to the Surgery group (17.8%) (HR 1.62, 95% CI: 1.01–2.61; *p* = 0.046). Both univariate and multivariate analyses confirmed the significantly higher loss‐to‐follow‐up rate in the AS group as well as in older patients. No deaths due to PTMC progression were observed in the cases lost to follow‐up.

**Conclusion:**

Despite concerns about loss to follow‐up, active surveillance remains a safe option for low‐risk PTMCs. Consistent follow‐up strategies are crucial, and further research is needed to enhance patient counseling and care for the management of patients with PTMC.

## Graphical abstract

What are the real‐world adherence rate and self‐discontinuation rate in papillary thyroid microcarcinoma (PTMC) patients undergoing active surveillance (AS) compared to those choosing surgical intervention? Among 327 low‐risk PTMC patients, 69.4% adopted AS of 2016. However, the AS group exhibited a notably higher 5‐year self‐discontinuation rate (28.6%) versus the Surgery group (17.8%), raising concerns about consistent adherence to AS protocols. Consistent follow‐up strategies are crucial for optimizing the management of patients with low‐risk PTMC.

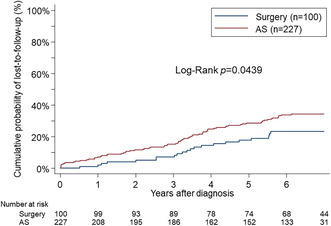

## 1 INTRODUCTION

Papillary thyroid microcarcinoma (PTMC) is a small, well‐differentiated thyroid cancer variant, typically ≤1 cm in diameter. Despite its excellent prognosis,^1^ determining the optimal management strategy for low‐risk cases remains uncertain. Traditionally, surgical resection, which may involve a total or partial thyroidectomy, has been the standard treatment for PTMC,^2^ but the recent recognition that not all PTMC cases require immediate surgery has brought a shift in the approach to PTMC management.^3^

Active surveillance (AS), a strategy involving the regular monitoring of PTMC without immediate surgical intervention, has gained prominence as a management option for patients with low‐risk PTMC. The AS approach is rooted in the understanding that PTMCs often follow an indolent course, and many patients with a PTMC may not experience significant disease progression during their lifetime. AS offers the advantage of avoiding surgical risks like hypothyroidism and vocal cord issues.

An AS policy for patients with low‐risk PTMC was first initiated in Japan and is now a widely acceptable management option nationwide.^4^ However, despite the growing acceptance of AS a suitable management option for low‐risk PTMC patients, studies conducted elsewhere, in such as the United States and Europe, have shown that the vast majority of PTMC patients in those regions opt for surgery instead of AS; indeed, AS has not gained widespread popularity outside of Japan.^5^, ^6^, ^7^, ^8^, ^9^ According to the data from the Surveillance, Epidemiology, and End Results (SEER) program, <1% of adult patients diagnosed with PTMC in the U.S. opted against surgery during the period 2000–2018.^5^

A key concern for both clinicians and patients is the potential for patient loss to follow‐up during AS, in which patients stop attending their regularly scheduled follow‐up visits and participating in monitoring. A study performed in the U.S. revealed that 78.4% of physicians cited the fear of patients becoming untraceable as a reason for not recommending AS,^6^ and follow‐up rates in real‐world settings are generally lower than those in intervention trials. This raises questions about the real‐world adherence rates and loss‐to‐follow‐up rates in PTMC patients choosing AS compared to those undergoing surgery.

We conducted the present study to investigate the real‐world adherence and loss‐to‐follow‐up rates in PTMC patients who opted for AS in comparison with PTMC patients who chose surgical intervention. Ito Hospital (Tokyo) specializes in the treatment of thyroid diseases and has long collected clinical data on PTC cases.^10^, ^11^, ^12^, ^13^, ^14^ For the present study, we used data from both Japan's National Cancer Registry Database and our hospital's medical records to assess the patient follow‐up and to evaluate factors influencing loss to follow‐up during AS. By examining the real‐world scenarios of both AS and surgical approaches, we seek to gain a deeper understanding of the challenges and advantages associated with them, as well as to assess the current status and issues regarding loss to follow‐up during outpatient visits.

## 2 MATERIALS AND METHODS

### 2.1 Study design and subjects

We selected the cases of patients who were diagnosed in 2016 with low‐risk PTMC via cytology, performed either at our hospital or by referring physicians, without any lymph node or distant metastases, and who subsequently underwent either surgical intervention or chose AS at our hospital. These patients were extracted from Japan's National Cancer Registry (NCR), which is a public cancer prospective registry in Japan that was launched in January 2016. Operated by a national institution, the National Cancer Center of Japan, the NCR consolidates, analyzes, and manages data from all individuals diagnosed with cancer in the country. Through this system, data from individuals diagnosed with cancer, regardless of their residential area or the healthcare institution where the diagnosis was made, are collected through cancer registration offices established in each prefecture and centrally managed in a national database. Municipalities submit vital information, including survival status and cause of death, using documents such as death certificates to the NCR as legally required. Therefore, the reliability of survival information in the NCR is high.

At our institution, we maintain a pathology database, which is systematically generated for prospective purposes. While surgical cases could be extracted comprehensively through this pathology database, cases involving the selection of AS presented a challenge. Given our institution's status as a specialized thyroid hospital, it is not rare for patients to receive a cytological diagnosis from referring physicians before being referred to our facility for treatment decisions. In such cases, when patients opt for AS, neither pathological nor cytological examinations are conducted at our institution, leading to an absence of such cases from our pathology database. For purposes of our study, which aimed to assess outcomes following surgical intervention or AS at our institution, it was impractical to rely solely on our institution's pathology database. Instead, we utilized NCR, a nationwide prospective registry, to extract cases in which our institution served as the treatment facility (regardless of the diagnosing institution), facilitating a more comprehensive analysis.

The data from the NCR were obtained in compliance with legal regulations and then further processed for our analyses. Patients diagnosed with PTMC at our hospital or by referring physicians but who underwent therapeutic procedures (i.e., surgery or AS) at other hospitals were excluded from the study. Approval to conduct this clinical study was granted by the Ito Hospital Institutional Review Board. We received the relevant data from the NCR on August 20, 2022.

### 2.2 Study variables

The patient data were primarily sourced from the NCR Database, which functions as a prospective registration database. Key patient characteristics including age, sex, distance from the hospital (calculated from the patients' home address information), mode of detection (by screening, incidental, unknown, or other), date of diagnosis, treatment method(s) (i.e., endoscopic resection, radiation therapy, chemotherapy, or unspecified) and patient survival status were derived from the NCR data.

For patient information that was not available in the registry, including the current outpatient status and tumor size, we conducted a retrospective review of the patients' medical records. We defined loss to follow‐up as a lapse in outpatient visits for >6 months beyond a scheduled follow‐up date, meaning patients who had not visited the clinic for over 6 months past their next scheduled appointment. For instance, for patients scheduled for a follow‐up visit in 6 months, we considered a lapse of over 1 year as indicative of loss to follow‐up. Similarly, for patients with scheduled appointments set for 1 year later, a lapse of over 1½ years without attendance would be classified as loss to follow‐up. Cases in which a patient was referred to other medical facilities to continue either AS or postsurgical follow‐up were not considered as instances of loss to follow‐up.

### 2.3 Survey on reasons for loss to follow‐up

A survey was conducted via phone interviews with patients who were lost to follow‐up. Cases in which a patient was referred to other medical facilities were not considered lost to follow‐up and were excluded from the survey. The survey asked patients whether they were currently receiving thyroid follow‐up at another facility. For those who were not, an open‐ended response was requested to identify the primary reason for discontinuation of treatment. After the open‐ended question, patients were asked to respond Yes or No to whether each of the following factors influenced their decision to discontinue treatment: lack of awareness of the possibility of cancer progression/recurrence, hospital distance, the COVID‐19 pandemic, a busy life, the burden of consultations/tests, and follow‐up costs.

### 2.4 AS and postoperative follow‐up protocol

Both postoperative and AS patients typically undergo check‐ups every 6 months. However, if patients remain stable, physicians can extend the interval between visits to 1 year. In AS, ultrasound examinations are conducted during each visit, following recommended protocols.^15^ These examinations are performed by ultrasound technicians, and the images are double‐checked by interpreting physicians and attending physicians.

### 2.5 Statistical analyses

Comparison of the loss to follow‐up rates between the AS and Surgery groups was performed using a Kaplan–Meier analysis and a Cox proportional hazards regression analysis. To analyze the determinants of loss to follow‐up, we examined a range of factors including therapeutic procedures, age, sex, distance from home to the hospital, and mode of detection. A p‐value threshold of <0.05 was considered significant. The statistical analyses were conducted using STATA software ver. 15.0 (Stata, College Station, TX).

## 3 RESULTS

### 3.1 Study population

A total of 327 patients with low‐risk PTMC without lymph node or distant metastases were enrolled. Among them, 100 patients underwent surgical intervention (Surgery group), and the other 227 patients opted for AS (AS group). Our hospital specializes in the treatment of thyroid diseases and has >100 thyroid specialists (including full‐time and part‐time physicians) who are actively involved in outpatient consultations. During the study period, a total of 110 endocrine surgeons and endocrinologists were involved in the examination and management of a subset of the enrolled patients.

Table 1 summarizes the clinicopathologic characteristics of the enrolled patients. The median age at diagnosis was similar in the Surgery (51 yrs) and AS groups (52 yrs). The sex distribution was also similar, with a majority being female in both the Surgery and AS cohorts (83% vs. 85%, respectively). Regarding the mode of PTMC detection, there was no significant between‐group difference. Among the four classifications of the mode of detection (by screening, incidental, unknown, or other), no patients within the study cohort were classified with an ‘unknown’ mode of detection.

**TABLE 1:** Baseline characteristics of the patients with low‐risk papillary thyroid microcarcinoma (PTMC) at the time of diagnosis.

	Surgery *n* = 100	AS *n* = 227	*p*
Age, yrs	51 (40, 64)	52 (40, 64)	0.971
Sex:			
Male	17 (17.0)	34 (15.0)	0.642
Female	83 (83.0)	193 (85.0)	
Distance from the hospital, km	19.1 (13.2, 37.5)	17.9 (9.4, 29.2)	0.195
Mode of detection:			0.067
By medical checkup	39 (39.0)	70 (30.8)	
Incidental discovery during follow‐up for other illnesses	30 (30.0)	99 (43.6)	
Other	31 (31.0)	58 (25.6)	

Abbreviation: AS, active surveillance.

### 3.2 Clinical pathways after diagnosis: Follow‐up and treatment changes

**FIGURE 1:**
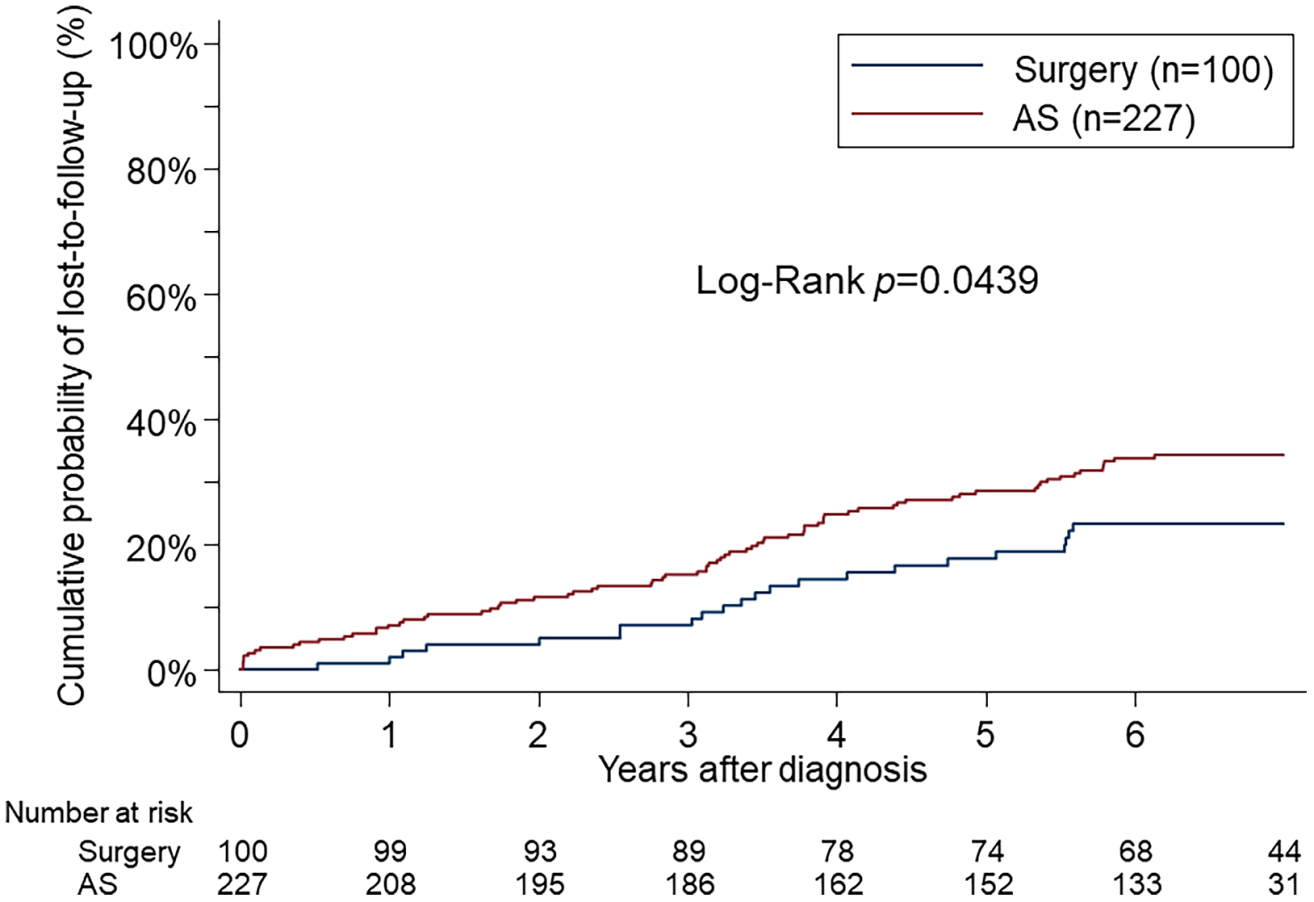
The Kaplan–Meier failure estimates of loss‐to‐follow‐up rates among patients with low‐risk papillary thyroid microcarcinoma (PTMC) who chose to undergo active surveillance (AS) or surgery. The loss‐to‐follow‐up rate was significantly higher in the AS group than in the Surgery group.

**FIGURE 2:**
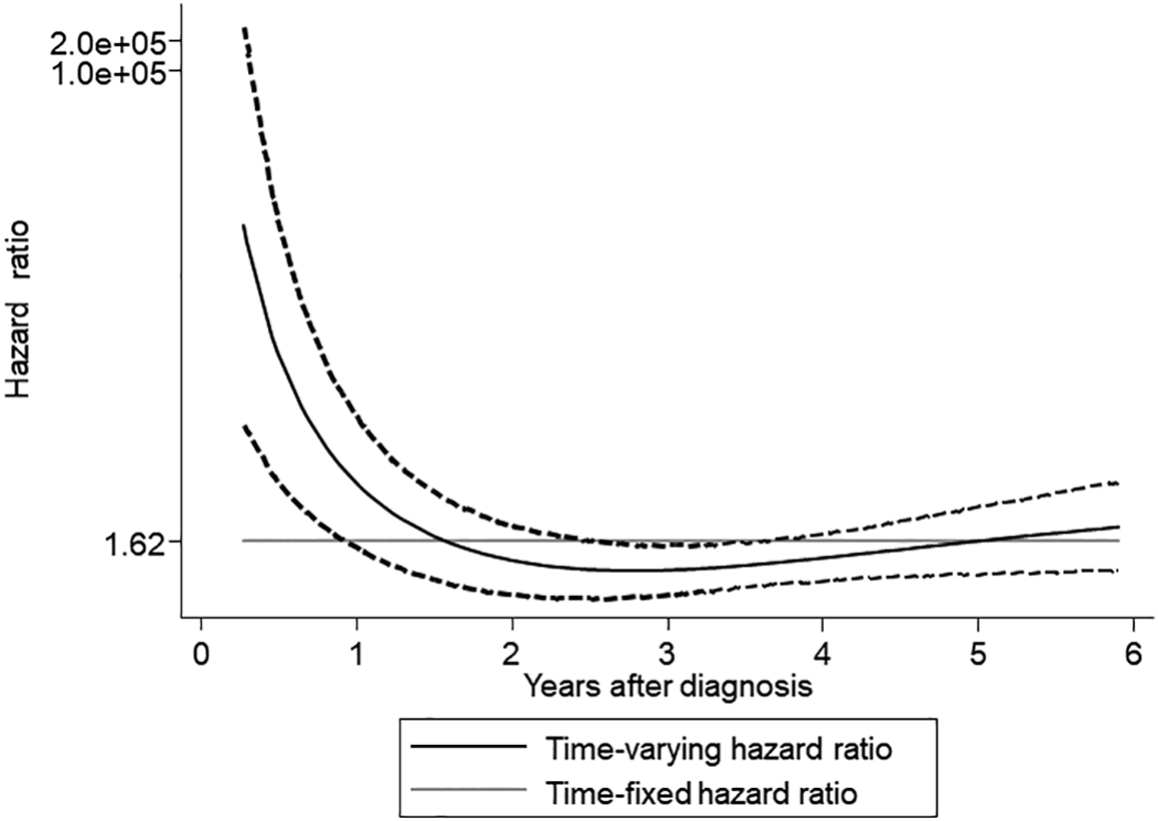
Time‐varying hazard ratios (HRs) for loss‐to‐follow‐up rates in low‐risk PTMC patients undergoing AS versus surgery. Over the interval of 0.5–2 years postdiagnosis, the HRs are relatively higher. Beyond the second year, the HRs show no significant changes.

The median follow‐up duration for the 327 enrolled patients was 6.45 years. The proportional hazard assumption was tested using the Schoenfeld residual and the log–log plot and found not to be violated. As depicted in Figure 1, the loss‐to‐follow‐up rate was significantly higher in the AS group compared to the Surgery group. The rates of loss to follow‐up at 3 and 5 years were 15.2% and 28.6% for the AS group and 7.1% and 17.8% for the Surgery group, respectively (hazard ratio [HR] 1.62, 95% confidence interval [CI]: 1.01–2.61, *p* = 0.046). No deaths were reported among the patients in the NCR database who self‐discontinued their AS or follow‐up. As shown in Figure 2, the time‐varying HRs for loss‐to‐follow‐up rates indicate that the HRs were relatively higher during the initial period postdiagnosis, suggesting the significant impact of treatment choice on loss to follow‐up. However, beyond the second year, the HRs stabilize, indicating a stabilization in the continuous impact of treatment choices over time.

During follow‐up, the cases of 19 of the 227 patients initially choosing AS transitioned to surgical intervention. The reasons for transitioning to surgery included tumor enlargement (*n* = 9 patients), emergence of other thyroid/parathyroid diseases requiring excision (*n* = 5; benign thyroid nodules [*n* = 3], Graves' disease [*n* = 1], and primary hyperparathyroidism [*n* = 1]), an additional consideration of surgical options due to a change in the attending physician (*n* = 3), and patient preference (*n* = 2). Of the two patient‐preference cases, one patient chose surgery after the appearance of intraglandular metastasis despite being given the option to continue AS, and the other patient opted for surgery after pregnancy and childbirth, considering future implications.

There were also nine cases in which surgery was deemed appropriate during AS but was not performed: three of these patients were considered for surgery due to the emergence of lymph node metastasis, and the other six patients were considered for surgery due to an increase in the size of the primary tumor. In three of the nine cases, AS was continued based on physician judgment, and in the other six cases, the patients opted against surgery despite their physicians' recommendations.

In the Surgery group, no instances of mortality or recurrence were observed during the study period.

### 3.3 Determinant analysis for loss to follow‐up

In Table 2, we present the outcomes of the simple and multiple logistic regression analyses exploring the factors influencing loss to follow‐up among patients diagnosed with low‐risk PTMC. Both the univariate and multivariate analyses revealed a significantly higher loss‐to‐follow‐up rate in the AS group, as well as in older patients. However, variables such as sex, distance from the hospital, and mode of detection showed no significant association with loss to follow‐up.

**TABLE 2:** Simple and multiple logistic regression models influencing the PTMC patients' lost‐to‐follow‐up.

	Simple logistic regression			Multiple logistic regression		
	OR	95% CI	*p*	OR	95% CI	*p*
Factors at the time of PTMC diagnosis						
Age	1.02	1.00–1.04	0.022*	1.02	1.00–1.04	0.021*
Sex	0.81	0.43–1.54	0.533	0.84	0.44–1.61	0.593
Distance from the hospital	1.00	1.00–1.00	0.123	1.00	1.00–1.00	0.100
Mode of detection	0.96	0.88–1.05	0.398	0.98	0.89–1.07	0.633
Factors after PTMC diagnosis						
Initial therapeutic procedure (surgery or AS)	1.75	1.01–3.02	0.045*	1.78	1.02–3.10	0.042*

*Note*: *p<0.05.

Abbreviations: CI, confidence interval, OR, odds ratio, PTMC, papillary thyroid microcarcinoma.

### 3.4 Survey results

**FIGURE 3:**
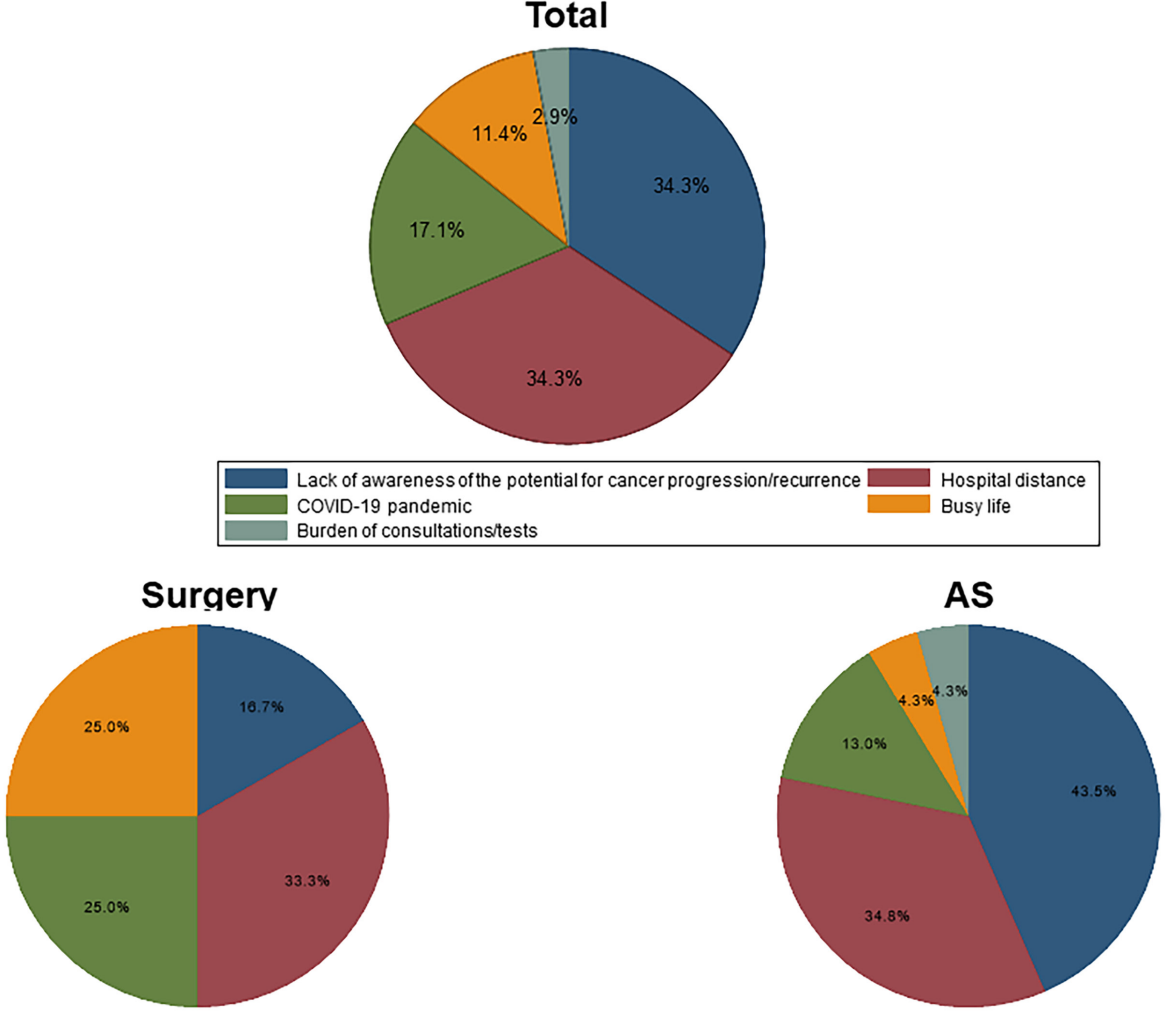
Primary reasons for loss to follow‐up. Distribution of primary reasons for loss to follow‐up, as identified by an open‐ended response from patients. The pie charts show the distribution of primary reasons among all patients, patients in the AS group, and patients in the Surgery group.

**FIGURE 4:**
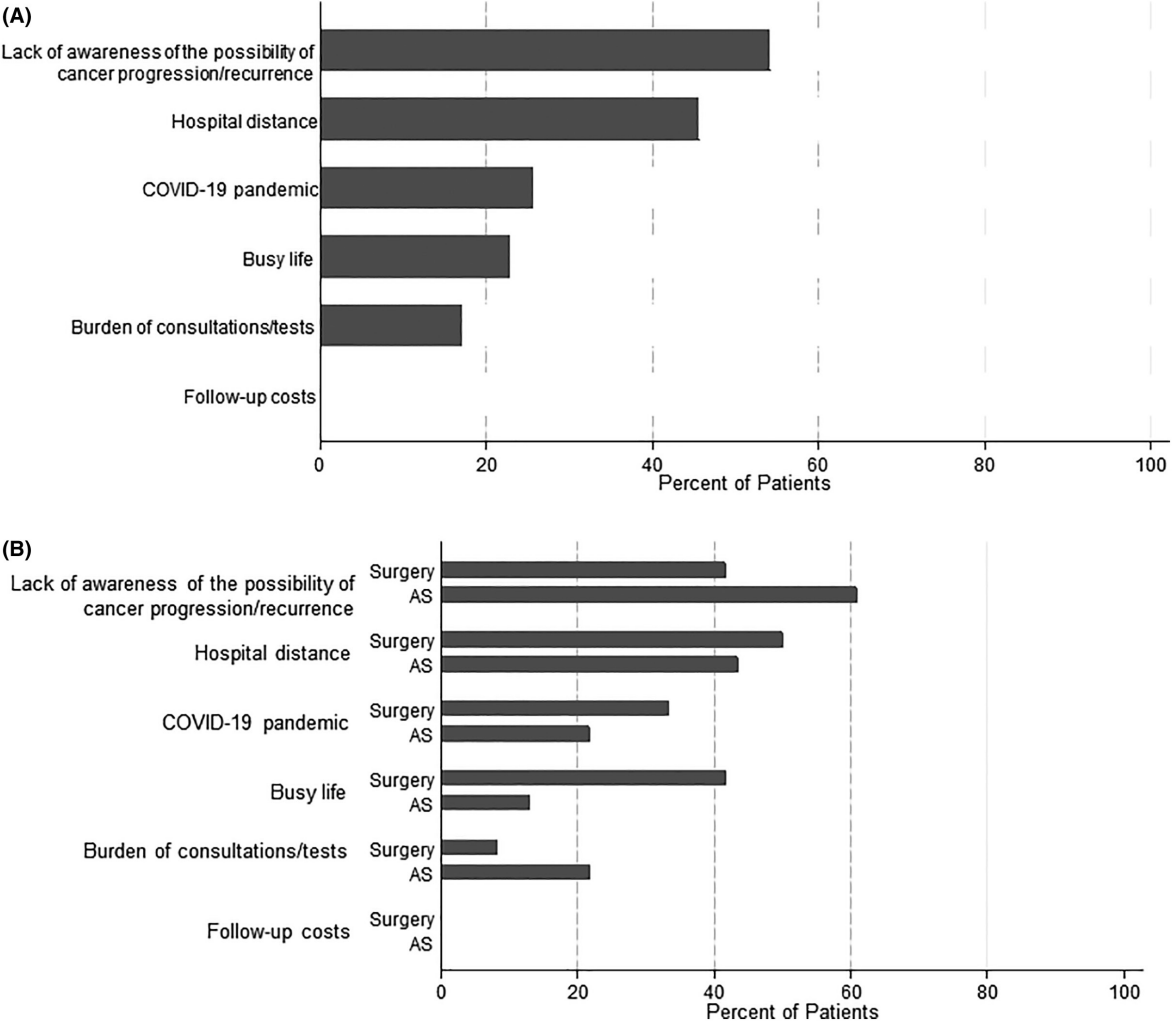
Factors influencing patients' decision to discontinue follow‐up. (A) Overall percentage of patients indicating whether each factor influenced their decision to discontinue follow‐up. (B) Percentage of patients in the AS and Surgery groups who responded “Yes” to whether each factor influenced their decision to discontinue follow‐up. Factors included a lack of awareness of the possibility of cancer progression/recurrence, hospital distance, the COVID‐19 pandemic, a busy life, the burden of consultations/tests, and follow‐up costs.

The loss‐to‐follow‐up rate during the study period was 22.0% (22 out of 100 cases) in the Surgery group and 33.0% (75 out of 227 cases) in the AS group. We attempted telephone interviews with all patients who were lost to follow‐up. We obtained responses from 12 out of 22 (54.5%) lost‐to‐follow‐up patients in the Surgery group and 29 out of 75 (38.7%) in the AS group. Among the AS group, six patients were currently visiting a local clinic without prior referral from our hospital. One patient, on their own initiative, sought a second opinion at another hospital and continued follow‐up there. Five patients transferred to a closer clinic due to distance issues, without any referral from our hospital. All six continued AS without transitioning to surgery. Excluding the six patients who transferred to another clinic, the survey results from 12 lost‐to‐follow‐up patients in the Surgery group and 23 in the AS group are presented in Figures 3 and 4.

Figure 3 illustrates the primary reason for loss to follow‐up, as identified through an open‐ended response. The pie charts present the distribution of primary reasons across the entire cohort, as well as separately for the AS and Surgery groups. The results indicate that the most common primary reason in the Surgery group was hospital distance (33.3%), followed by busy life (25.0%) and the COVID‐19 pandemic (25.0%), while that in the AS group was lack of awareness of the possibility of cancer progression (43.5%), followed by hospital distance (34.8%). Regarding the lack of awareness of the possibility of cancer progression (for the AS group) or recurrence (for the Surgery group), some patients in the AS group stated that they were not concerned about progression due to the tumor size remaining stable during their visits, leading to a decreased perception of the potential for cancer progression and cancer‐related anxiety. In the Surgery group, some patients believed that after 5 years without recurrence, their risk of cancer returning was minimal, similar to other cancers. Among the 12 patients who cited hospital distance, five reported physical reasons such as difficulty in traveling due to worsening conditions like leg issues, four had moved further away, two had always found the hospital too far from their homes since diagnosis, and one had initially found the hospital convenient due to proximity to their workplace but found it too far away after leaving the job.

Figure 4 shows the distribution of reasons given for loss to follow‐up among the surgery and AS groups. The loss‐to‐follow‐up patients were asked whether each of 6 items impacted loss to follow‐up in their cases and responded Yes or No for each: lack of awareness of the possibility of cancer progression (for the AS group) or recurrence (for the Surgery group), hospital distance, the COVID‐19 pandemic, busy life, the burden of consultations/tests, and follow‐up costs. In the AS group, the distribution of responses was similar to the primary reasons in Figure 3, with a high percentage of patients not worried about cancer progression. In the Surgery group, although there was no significant difference, more patients cited multiple reasons compared to the AS group (75.0% vs. 43.5%). As a result, the distribution of reasons shown in Figure 3 differed slightly from the distribution in Figure 4 for the Surgery group. Additionally, though not statistically significant, a higher percentage of the Surgery group cited a busy life compared to the AS group (41.7% vs. 13.0%), while a higher percentage of the AS group cited the burden of consultations/tests compared to the Surgery group (21.7% vs. 8.3%). In neither group did any patient indicate that follow‐up costs influenced their decision to discontinue follow‐up.

## 4 DISCUSSION

We investigated real‐world loss‐to‐follow‐up rates and safety considerations in Japanese PTMC patients who opted for AS or surgical intervention, and our findings provide several important insights into the clinical pathways, follow‐up rates, and safety profile among patients who chose AS the management strategy for their low‐risk PTMC. The most notable finding highlighted in this study is the substantial prevalence of AS adoption among low‐risk PTMC patients in routine clinical practice (not clinical trials), amounting to 69.4% (227 of 327 low‐risk PTMC cases) as of 2016. As the data from the SEER program indicated, fewer than 1% of adult patients with PTMC have opted out of surgery between 2000 and 2018 in the U.S.^5^ These data and those obtained in other studies illustrate the much lower utilization of AS in countries other than Japan,^5^, ^6^, ^7^, ^8^, ^9^ limited to extremely select patients even in low‐risk PTMC. Conversely, in Japan, AS is outlined in clinical guidelines^16^, ^17^ and is commonly chosen as a viable treatment option for PTMC. At our hospital, we present both AS and surgical intervention as choices in the majority of low‐risk PTMC cases, allowing patients to select their preferred option. The AS group in the present study thus encompassed a broader patient spectrum that was not composed solely of highly selective cases.

The results of our analyses revealed a notably higher loss‐to‐follow‐up rate in the patients undergoing AS compared to those who chose surgical intervention. This finding underlines a crucial concern regarding the consistent adherence of PTMC patients to AS protocols. As described in a survey report by Hughes et al., 78.4% of physicians cited the fear of patients becoming untraceable as a reason for not recommending AS.^6^ Consistent with their concerns, the real‐world data in the present study indicated a higher proportion of patients becoming untraceable in the AS group compared to the Surgery group. It could thus be concluded that the importance of consistent follow‐up visits cannot be overstated to patients who opt for AS, as discontinuation might result in missed opportunities for the early detection of disease progression. However, the findings of this study also demonstrated that among the individuals who self‐discontinued outpatient visits, there were no reported deaths recorded in the NCR database. Those patients' loss to follow‐up did not lead to mortality due to PTMC progression, indicating that even with some loss to follow‐up, AS can still be considered a safe treatment choice for PTMC, considering the excellent overall prognosis for PTMC.^1^ However, the lack of fatalities should by no means be misconstrued to indicate that continued surveillance is unnecessary. While the study was able to confirm the vital status of patients who discontinued follow‐up, it did not assess the progression of PTC. There may be cases in which surgery needs to be considered during AS due to tumor enlargement or lymph node metastasis, albeit infrequent.^18^ Therefore, the continuation of surveillance remains essential, as the criteria for identifying patients who may require surgical intervention during AS are not yet well‐known.

We also conducted an analysis of factors related to loss to follow‐up. To the best of our knowledge, there is a lack of reported risk factors specifically associated with loss to follow‐up in the AS of PTMC. In other diseases, factors such as age,^19^, ^20^, ^21^ sex,^20^, ^21^ and residing a long distance from the hospital^22^ have been reported as risk factors for loss to follow‐up. Although our results indicated that older age was a potential risk factor for loss to follow‐up, we found no significant indication of sex or distance from the hospital as risk factors. For patients opting for AS and elderly individuals, it may be crucial to thoroughly explain the necessity of regular visits and provide information about the possibility of referral to nearby medical facilities if attending appointments becomes difficult in the future.

Additionally, recognizing the limitations of interpreting data solely from existing records, we conducted telephone interviews with patients who were lost to follow‐up. From these interviews, we found that a high percentage of patients in the AS group who discontinued follow‐up did not perceive any risk of cancer progression, with many stopping visits solely for this reason. Some patients noted that the lack of change in tumor size during follow‐up led to a reduction in cancer‐related anxiety, suggesting the need to continuously communicate the importance of regular monitoring during outpatient visits, even when there are no changes in tumor size. There have been cases reported in which tumors remained stable for over 15 years before showing growth,^23^ indicating that it is not yet established which cases can be considered safe to discontinue follow‐up. Therefore, ongoing follow‐up should be recommended, even when there are no changes in tumor size. In this regard, exploring educational interventions for patients and healthcare providers to emphasize the importance of regular monitoring could be beneficial. Furthermore, no patients indicated that follow‐up costs influenced their decision to discontinue follow‐up. Given Japan's national health insurance system, where medical expenses are generally covered, patients may not perceive follow‐up costs as a factor contributing to discontinuation of follow‐up. The impact of follow‐up costs could vary depending on the healthcare system of each country.

In Japan, the coronavirus disease 2019 (COVID‐19) pandemic began in 2020,^24^ falling within the timeframe of our study, corresponding to the fourth year after the diagnosis of low‐risk PTMC. Although the occurrence of the COVID‐19 pandemic during the study period raised concerns about its impact on discontinuation of outpatient visits, there was no significant increase in loss to follow‐up attributable to the COVID‐19 pandemic (Figure 1). As shown in Figure 2, the HRs tended to stabilize beyond 2 years postdiagnosis, indicating consistent trends unaffected by the Covid‐19 pandemic. This suggests that even if the pandemic had an influence, it did not disproportionately affect either the AS or Surgery groups. Moreover, the proportional hazard assumption was not violated, indicating that the pandemic did not have a substantial effect on the proportional hazards for either the AS or Surgery groups. Furthermore, as shown in Figure 3, the proportion of patients citing the COVID‐19 pandemic as a reason for loss to follow‐up in the telephone interviews was limited. Throughout the study period, both groups exhibited a consistent occurrence of loss to follow‐up, suggesting that the impact of the COVID‐19 pandemic on loss to follow‐up may have been minimal in our study.

While specific risk factors associated with loss to follow‐up in the AS of PTMC remain elusive, understanding and addressing potential barriers to consistent follow‐up may be imperative for optimizing patient care.

## 5 STUDY LIMITATIONS

Some study limitations should be noted: (1) the incomplete coverage of risk factors for loss to follow‐up; (2) the single‐center nature of the study; and (3) the lack of analysis regarding the decision‐making process for AS or surgical intervention. First, the analyses we conducted did not comprehensively cover all risk factors associated with loss to follow‐up. In other diseases, factors beyond those investigated herein, such as the patient's perception that treatment was no longer required,^25^ a planned or current pregnancy,^25^ family income,^21^ smoking,^21^ and treatment by nonspecialists,^19^ have been reported as potential risk factors for loss to follow‐up. However, these data are not included in the NCR database, limiting our ability to identify all of the risk factors contributing to loss to follow‐up in this study. Therefore, in light of the results of this study, it is important to highlight that the higher dropout rate in the AS group should not automatically lead to the conclusion that immediate surgery is the preferred option. Various confounding factors, such as patient backgrounds and reasons for choosing AS, may have contributed to the higher dropout rate in the AS group compared to surgery. To further improve the loss‐to‐follow‐up rate, it is crucial to address other known risk factors from different diseases that were not included in the analysis. For instance, during telephone interviews, the primary reason identified for loss to follow‐up among AS cases was the patient's perception that treatment was no longer required. Therefore, it is crucial to thoroughly explain the possibility of a need for surgery arising during AS to address this perception.

Second, data from a single thyroid‐specific hospital were used in this study. The results may not be fully representative of other populations or healthcare settings. Last, we did not analyze the decision‐making process of the patients who opted for AS or surgical intervention. Factors such as nuances during treatment recommendations by primary physicians or patients' social backgrounds, which cannot be obtained from the NCR database, intricately intermingle in the treatment selection process. Evaluating such aspects would ideally involve prospective surveys directly gathering insights from patients.

In conclusion, the present study illuminates real‐world adherence and loss‐to‐follow‐up rates in low‐risk PTMC patients undergoing AS versus surgery, providing insights into clinical pathways. Despite substantial AS adoption in Japan, higher loss‐to‐follow‐up rates raise adherence concerns. Regardless, no PTMC progression‐related deaths occurred among AS patients who self‐discontinued, suggesting the relative safety of this approach. However, given the potential for tumor progression, consistent follow‐up strategies are vital for optimizing low‐risk PTMC management.

## AUTHOR CONTRIBUTIONS

**Yoshiyuki Saito:** Conceptualization (equal); formal analysis (lead); investigation (equal); writing – original draft (lead). **Kenichi Matsuzu:** Conceptualization (equal); investigation (equal); writing – review and editing (equal). **Hiroshi Takami:** Conceptualization (equal); validation (equal). **Ai Matsui:** Validation (equal). **Yoko Kuga:** Validation (equal). **Ryoji Ohara:** Validation (equal). **Kana Yoshioka:** Validation (equal). **Chie Masaki:** Validation (equal). **Junko Akaishi:** Visualization (equal). **Kiyomi Y. Hames:** Validation (equal). **Ritsuko Okamura:** Validation (equal). **Chisato Tomoda:** Validation (equal). **Akifumi Suzuki:** Validation (equal). **Wataru Kitagawa:** Validation (equal). **Mitsuji Nagahama:** Validation (equal). **Kiminori Sugino:** Methodology (equal); validation (equal). **Koichi Ito:** Supervision (equal); validation (equal).

## FUNDING INFORMATION

No funding was received for this article.

## CONFLICT OF INTEREST STATEMENT

The authors have no conflict of interest to report.

## ETHICS STATEMENT

Approval to conduct this clinical study was granted by the Ito Hospital Institutional Review Board. Information disclosure was conducted in accordance with Japanese laws governing the NCR, which mandates an opt‐out format (written informed consent is not required).

## CONSENT

Verbal telephone consent was obtained for loss‐to‐follow‐up patients.

## Data Availability

The data that support the findings of this study are available on request from the corresponding author. The data are not publicly available due to privacy or ethical restrictions.
